# Supplementary material for: Cytoplasmic p53β Isoforms Are Associated with Worse Disease-Free Survival in Breast Cancer
Source: Int J Mol Sci. 2022 Jun 15;23(12):6670. doi: 10.3390/ijms23126670 (PMC9223648; doi:10.3390/ijms23126670)
Supplement: Supplementary file 1 [file ijms-23-06670-s001.zip › ijms-1756963-supplementary.pdf]

Supplementary

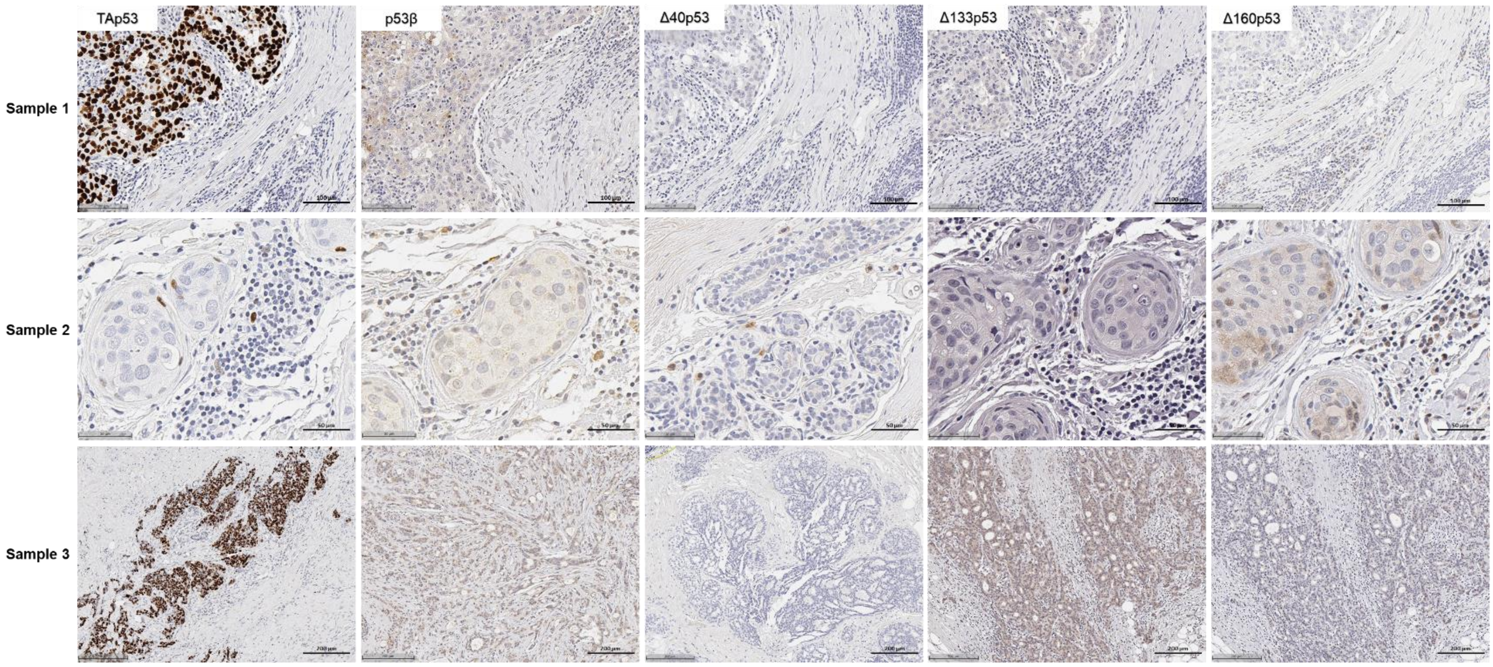

Supplementary Figure S1: Representative images of the three specimens stained for TAp53 (DO-1), p53β (KJC8), Δ40p53 (KJC40), Δ133p53 (KJC133), or Δ160p53 (KJC160). Scale bar represents 100 μm (sample 1), 50 μm (sample 2), and 200 μm (sample 3).

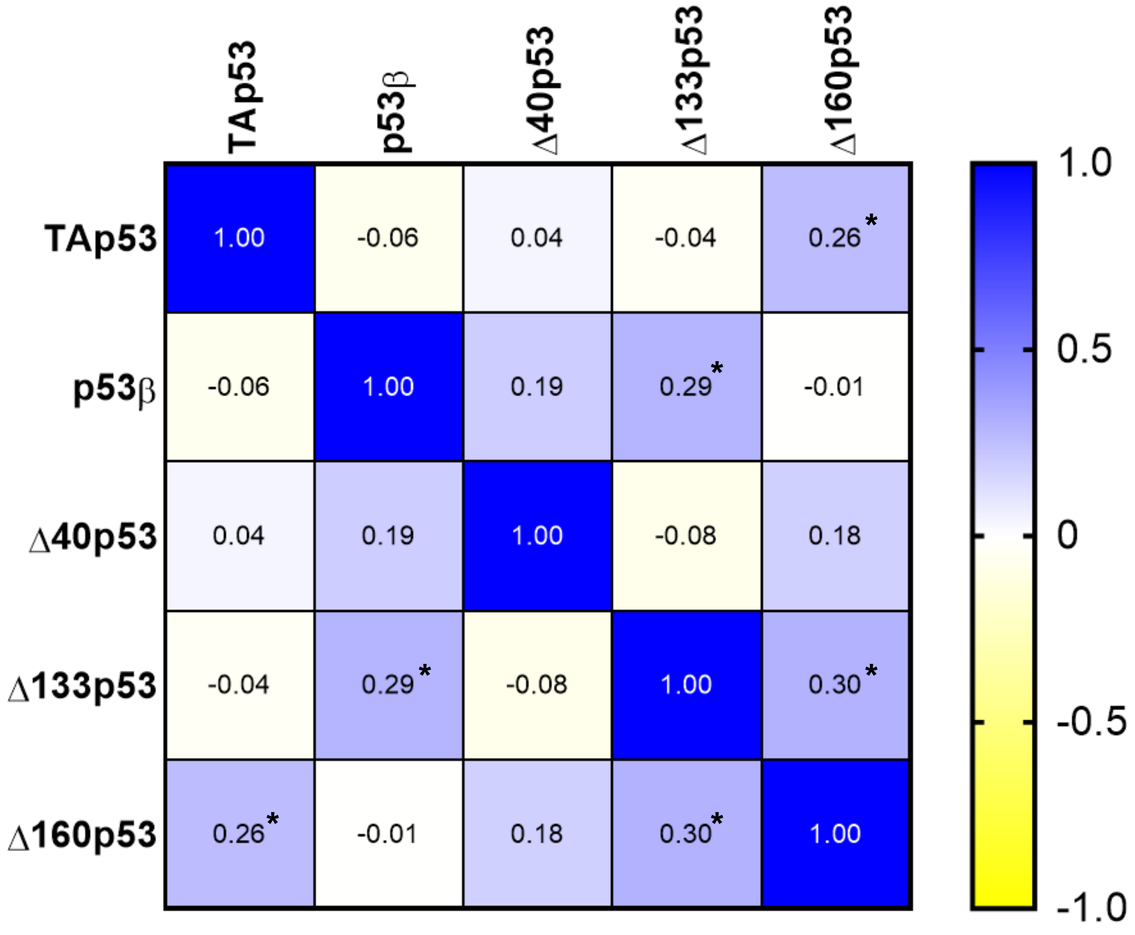

Supplementary Figure S2: Correlation of p53 isoform expression with one another. Correlation analyses were performed using the Spearman rank correlation coefficient to evaluate the relationship between individual p53 isoform expression in 108 breast cancer specimens. p53β, and Δ133p53, and Δ133p53 and Δ160p53 were found to be significantly correlated with one another. Results were considered significant at $p < 0.05$ (*).

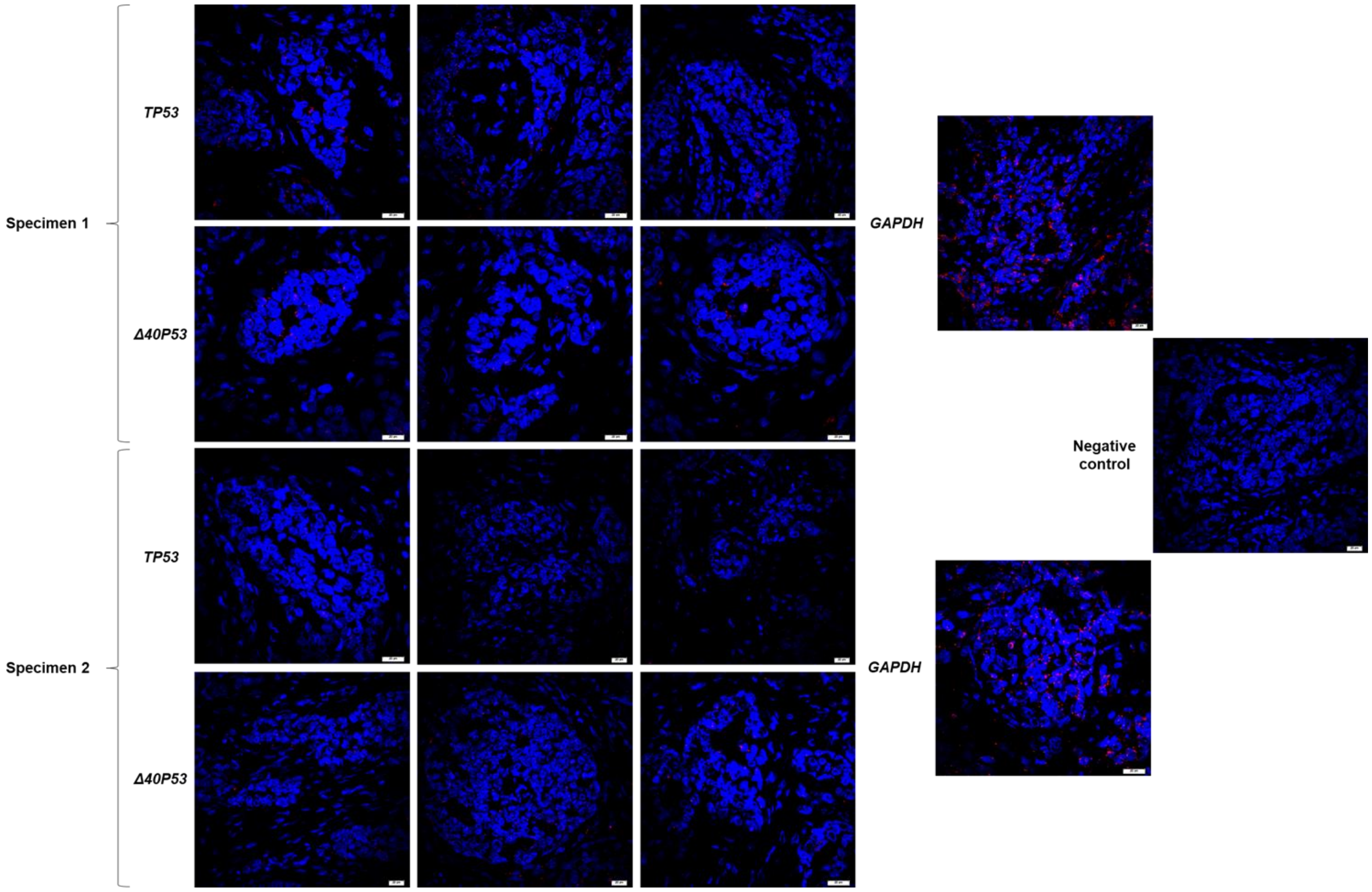

Supplementary Figure S3: *In situ* hybridisation of two TNBC specimens for *Δ40p53* or *TP53* (shown in red). *GAPDH* was used as a positive control. Cells nuclei were stained with DAPI. Scale bar represents 20 µm.

Supplementary Table S1: Association of p53 isoform expression and clinicopathological features.

Isoform	Clinicopathological features	Nuclear staining *p* value	Cytoplasmic staining *p* value
TAp53	Age	0.2254	0.6241
	Grade	0.9247	0.9312
	Tumour size	0.9918	**0.0329**
	LN+	0.3945	0.9620
	ER+	0.1404	0.1047
	PR+	0.3810	0.4354
	HER2+	0.0522	0.3170
	TP53 mut	**0.0022**	0.0963
p53β	Age	0.4676	0.6803
	Grade	0.0995	0.9759
	Tumour size	0.2898	0.2787
	LN+	0.4302	0.3778
	ER+	0.3623	0.3609
	PR+	0.7746	0.9952
	HER2+	0.6843	0.6124
	TP53 mut	0.3326	0.639
Δ133p53	Age	0.437	0.7112
	Grade	0.7186	0.8194
	Tumour size	0.6717	0.3409
	LN+	0.8604	0.8893
	ER+	0.3144	0.2218
	PR+	0.3627	0.3743
	HER2+	0.7816	0.2178
	TP53 mut	0.7462	0.3182
Δ160p53	Age	0.4358	0.0995
	Grade	0.1831	0.0835
	Tumour size	0.2862	0.5424
	LN+	0.2235	0.2082
	ER+	0.1995	0.3808
	PR+	0.3203	0.3274
	HER2+	0.5023	0.5694
	TP53 mut	0.9489	0.1763

Multiple linear regression was used to determine statistical significance. Results were considered significant at $p < 0.05$. LN: lymph nodes; ER: oestrogen-receptor; PR: progesterone receptor; HER2: human epidermal growth factor receptor 2.
